# Uptake of HIV testing among adolescents and associated adolescent-friendly services

**DOI:** 10.1186/s12913-020-05731-3

**Published:** 2020-09-17

**Authors:** Rachel Kidman, Jennifer Waidler, Tia Palermo, On Behalf of the Tanzania Adolescent Cash Plus Evaluation Team, Tia Palermo, Valeria Groppo, Lusajo Kajula, Jacobus de Hoop, Leah Prencipe, Jennifer Waidler Johanna Choumert Nkolo, Respichius Mitti, Bhoke Munanka, Paul Luchemba, Tumpe Mnyawami Lukongo, Aroldia Mulokozi, Ulrike Gilbert, Paul Quarles van Ufford, Rikke Le Kirkegaard, Frank Eetaama

**Affiliations:** 1 Department of Family, Population and Preventive Medicine, HSC Level 3, Room 79 Stony Brook University (State University of New York), Stony Brook, NY 11794 USA; 2 UNICEF Office of Research – Innocenti, Via degli Alfani 58, 50121 Florence, Italy; 3 Department of Epidemiology at Environmental Health, 270 Farber Hall, University at Buffalo (State University of New York), Buffalo, NY 14214-8001 USA

**Keywords:** Adolescents, Tanzania, HIV testing, SRH, Adolescent-friendly services

## Abstract

**Background:**

HIV testing remains low among adolescents. Making public health services more adolescent-friendly is one strategy used to encourage testing. However, it remains unclear whether government-led initiatives have a meaningfully impact.

**Methods:**

The current study is observational and utilizes two sources of data (health-facility and adolescent-level) from one round of data collection of an on-going, longitudinal impact evaluation of a pilot cash plus program targeting adolescents. This study linked data from adolescent surveys (*n* = 2191) to data collected from nearby government-run health facilities (*n* = 91) in two rural regions of Tanzania. We used log binomial regression models to estimate the association between specific adolescent-friendly health service (AFHS) characteristics and adolescents’ uptake of 1) HIV testing and 2) visiting a health care facility in the past year for sexual and reproductive health (SRH) services.

**Results:**

Most adolescents (67%) lived in a village with a health facility, and all offered HIV services. We find, however, that AFHS have not been fully implemented. For example, less than 40% of facilities reported that they had guidelines for adolescent care. Only 12% of facilities had a system in place for referral and follow-up with adolescent clients, yet this was an important predictor of both past-year HIV testing (RR = 1.28, *p* < 0.1) and SRH visits (RR = 1.44, *p* < 0.05). Less than half (44%) offered services for survivors of gender-based violence (GBV), a significant predictor of past-year HIV testing (RR = 1.20, p < 0.05) and SRH visits (RR = 1.41, *p* < 0.01) among sexually-active adolescents.

**Conclusions:**

We find that national guidelines on AFHS have not been fully translated into practice at the local level. We highlight particular gaps in adolescent referral systems and GBV services. Scaling up these two essential services could encourage greater HIV testing among a high-risk population, in addition to providing much needed support for survivors of violence.

## Background

Almost four million youth (age 15–24) were living with HIV at the end of 2017, with over half a million newly infected that year [1]. HIV testing is a critical entry point for both prevention and treatment [2]. However, only a minority of adolescents have ever been tested [3]. In the United Republic of Tanzania, for example, less than half of female youth with HIV infection are aware of their status [4]. Low rates of testing result in late diagnosis, delayed care, and high mortality among youth [3, 5]; it also results in increased mother-to-child transmission [6].

Increasing adolescent testing is a clear priority. However, most HIV interventions focus on individual barriers, and don’t pay adequate attention to health system barriers where solutions are most scalable (for example, [7]). While a 2013 World Health Organization (WHO) study found fear was the primary barrier to testing for adolescents, they also cited important service-delivery factors: difficulty accessing HIV testing services, a lack of appropriate adolescent services, concerns about confidentiality, and poor provider attitudes [8]. Thus, it may be possible to increase uptake of vital services – such as HIV testing – by ensuring health facilities are in locations accessible to adolescents, are open after school hours, offer comprehensive sexual and reproductive health (SRH) services, ensure privacy, and are delivered in an adolescent-friendly manner by providers.

International efforts have focused on making public health services more adolescent-friendly. Almost two decades ago, the WHO issued recommendations for HIV services to be based around five adolescent-friendly principles: they should be accessible, acceptable, equitable, appropriate and effective [9]. In 2005, Tanzania’s Ministry of Health and Social Welfare (MOHSW) responded by issuing “Standards for Adolescent Friendly Reproductive Health Services.” The MOHSW has since worked to create a shared understanding of AFHS and to support quality implementation at health facilities [10]. Adolescent-friendly health services (AFHS) remain a key component of national HIV responses, both globally and in Tanzania [11–14].

Despite substantial investment, there is very little evidence that the AFHS strategy is working. A systematic review of interventions to improve adolescent access to SRH services in low- and middle-income countries (LMIC) found mixed results [15]. There are some positive findings: In Malawi, for example, adolescents were more likely to access HIV testing and three times more likely to receive condoms at a health clinic with AFHS (id est., with a youth space, longer hours, peer educators) [16]. In Tanzania, a cluster randomized control trial (RCT) of an AFHS intervention reported improvements in the quality of staff interactions. However, the Tanzanian intervention had only a modest impact on male utilization of STI services, and no impact on female utilization or sexual health outcomes [17, 18]. A review concluded that the strongest evidence supports a multipronged approach: simultaneously making adolescent-friendly adjustments to the facilities, training health care workers, and implementing community outreach to increase acceptance of adolescent SRH services. The authors recommend further research to understand which specific AFHS components were necessary for impact [15].

Moreover, much of the previous research focuses on intensive clinical interventions (for example, creating adolescent-focused spaces and staffing peer educators) that make the facility more responsive to adolescent needs. Even if AFHS are efficacious in researcher-led initiatives, there is no guarantee that they will be implemented with high fidelity and have the same impact in when scaled up nationally. South Africa was at the forefront of this effort, rolling out an Adolescent-Friendly Clinic Initiative in 1999 [19]. Subsequent assessments have found that few facilities meet the criteria for AFHS outlined by national guidelines [20], and evidence for its impact is limited [21].

In Tanzania, it remains unclear whether government-led AFHS initiatives have had a meaningfully effect on adolescent service utilization. Almost a decade after introducing AFHS standards, studies documented significant implementation challenges. Many clinics were still not aware of the guidelines, not implementing AFHS, and did not have providers with adolescent SRH knowledge [10, 22, 23]. In a study of 33 government health facilities, mystery clients reported limited hours, a lack of STI services, inadequate privacy, and negative attitudes towards adolescent SRH services use [23]. Importantly, none of these studies examined the impact of the nationally-scaled AFHS on health behaviors, such as HIV testing.

This study describes the availability of AFHS, adolescents’ perception of care, and adolescents’ utilization of services in two rural regions of Tanzania. The literature summarized above generally does one of two things: 1) broadly assesses adolescent-friendliness of services offered or 2) examines changes in uptake after specific, labor-intensive interventions. In terms of the latter, the evidence suggests that measures such as youth waiting rooms and peer educators have limited effectiveness [24], and these efforts may not be easily scalable. In the current study, we contribute to the literature by linking data on quality of health services in existing government facilities to individual-level, adolescent self-reported services utilization data from household surveys to examine whether the availability of AFHS leads, as expected, to greater utilization of health services. Thus, this study estimates the association of specific AFHS factors with adolescent uptake of HIV testing and SRH service use. We hypothesize that the more adolescent-friendly and accessible the nearby clinic, the more likely respondents will be to report past-year HIV testing and SRH service use.

## Methods

### Study setting

The current study uses data collected as part of an on-going, longitudinal impact evaluation of a pilot entitled a “Cash Plus” Model for Youth Well-Being and Safe, Healthy and Productive Transitions to Adulthood. The study was implemented in four districts: Rungwe and Busokelo in Mbeya region and Mufindi and Mafinga in Iringa region. Mbeya is located in the South West Highlands, while Iringa is in the Southern Highlands zone. In these regions, agriculture is the most common sector of employment, and the populations face significant health challenges, including higher than national averages of HIV infection, high rates of stunting and low rates of access to health insurance [25].

Financing for health services in Tanzania relies on a mixture of taxes and user fees. There are three main insurance schemes introduced by the MoHSW, including the Community Health Fund, TIKA (stands for Tiba Kwa Kadi and translates to “treatment with card”), and the National Health Insurance Fund [26]. CHF requires an annual premium, even for extremely poor households, and NHIF is available only to formal sector employees. Nationally, the rates of uninsured are 91% among women and 90.5% among men. In Iringa and Mbeya, specifically, the rates are 89.7 and 90.4 (compared to 88.8 and 84% among men), respectively [27]. There are fees associated with most health services in Tanzania, however some services, such as HIV testing and treatment, are provided free to the public through government subsidization. Some facilities may opt to use a sliding scale fee and take into account ability to pay in determining fees.

### Study design

This study is observational, but uses secondary data on adolescents and health facilities gathered during an impact evaluation. The evaluation utilizes cluster randomized control trial (cRCT) design to assess an intervention implemented by the Tanzania Social Action Fund (TASAF), an agency of the Government of the United Republic of Tanzania. The intervention targeted adolescents living in extremely poor households participating in the government’s flagship social protection program, the Productive Social Safety Net (PSSN). Components of the adolescent-focused intervention included: 1) livelihoods and life skills training; 2) mentoring and a productive grant; and 3) linkage to strengthened HIV, SRH and violence response services provided by government-run, primary health care facilities. More information on the overall impact evaluation study design and sampling is provided in the Appendix.

The current observational study leverages data from the second round of data collection of this on-going impact evaluation, which follows a panel sample of 2191 youth from 1779 households in 130 villages. Eligibility criteria for youth to be included in the study were as follows: 1) aged 14–19 years at baseline and 2) living in a PSSN household at baseline. Thus, all study households receive cash transfers and other components of the PSSN. Baseline surveys were conducted between April and June 2017, prior to randomization of villages into study arms. The adolescent-focused intervention was randomized at the village-level (65 randomized to treatment and 65 randomized to control). Round 2 of the youth/household surveys was conducted May – July 2018. Youth questionnaires were administered directly with youth, using same-sex enumerators. Informed consent was obtained from all youth aged 18 years and above and for married youth aged 14 to 17 years. For unmarried adolescents aged 14 to 17 years, informed consent was obtained from household head or caregiver, and informed assent was obtained from the youth. Questionnaires were administered in private areas in and around the household, where family members or others could not overhear sensitive topics of the questionnaire.

This study also includes data from 91 government-run, primary health care facilities. Eligibility criteria for inclusion of health facilities in the study were: 1) government-run primary health care facility, including dispensaries and 2) located in study area. Health facility surveys were administered to staff working at these facilities. Data used in the current analysis come from Round 2 of the health facility data collection (conducted February–March, 2018). For more information on sample selection and data collection, see Tanzania Cash Plus Evaluation Team (2018) [28].

To link the facility- and adolescent-level data, adolescents were assigned to a health facility based on their residential location. A third (33%) of the adolescents had no health facility located in their village. In these cases, the adolescent was matched to the closest health facility in another village based on GPS coordinates. About a quarter of adolescents (26%) had more than one health facility located in their village. These adolescents were assigned to the facility that reported performing the highest number of adolescent HIV tests in the past year. The rationale for this choice was that adolescents as a group had already indicated, through their choice of clinic, where they were most likely to seek HIV testing. While there were 91 health facilities in the data, only 69 resulted in the closest match for adolescents in the study sample, and thus *n* = 69 facilities comprises the sub-sample for analyses.

### Measures

*The main independent variables of interest related to availability of adolescent-friendly services*. Table 2 lists the variables used to define the indicators, organized by the WHO’s five objectives for assessing quality of health care services for adolescents. To examine whether health facilities are accessible to adolescents, for example, we measured whether they are conveniently located, had adequate opening hours, offered affordable services, made efforts to inform adolescents about their health services, and tried to communicate the importance of adolescent services to the community. Health facility surveys also captured indicators of whether services were equitable (for instance, provided to all adolescents irrespective of their marital status), appropriate (e.g., offered comprehensive SRH), and effective (e.g., availability of adolescent friendly trained staff, use of evidence-based protocols).

The final WHO quality objective – the acceptability of services – can only be assessed from the perspective of the adolescent. Those adolescents who reported utilizing SRH services were thus asked about whether they felt comfortable asking SRH staff questions; whether staff answered SRH questions adequately; whether staff were friendly, and whether SRH services were adequately confidential. Since such indicators were only available for a subset of the adolescents and had high endorsement, we do not include these factors in models predicting uptake of HIV and SRH services.

*The main outcome of interest was utilization of health services*, categorized with two indicators: 1) HIV testing and 2) visiting a health care facility in the past year for HIV/SRH services. HIV testing was captured using the questions, “I do not want to know the result, but have you ever been tested for HIV?” If the answer was yes, a follow-up question was asked about testing behavior in the past year. While HIV testing can take place at community organizations or even at home, other SRH services are primarily accessible through health facilities. Adolescents were asked if they had visited a health facility in the past year for HIV/SRH services, defined as services related to contraception, pregnancy, or sexually-transmitted infections (STI) testing or treatment.

*Control variables used in the regression analyses include* basic health facility characteristics (type of clinic (health care facility v. dispensary), number of medical staff) and adolescent demographic characteristics (gender, age, education, marital status, and sexual debut).

### Analysis

Frequencies and means (as appropriate) are used to characterize the adolescent sample, the health facilities, and the availability of AFHS. To estimate the association between facility-level AFHS characteristics and adolescents’ uptake of services, we calculated risk ratios using multivariate log binomial regression models. One regression was separately run for each independent variable (indicating facility-level quality), and standard errors were clustered at the village level to account for the fact that all adolescents within one village are linked to the same health facility. All models include the control variables listed above. For both past-year outcomes, we also created models stratified by whether the adolescent reported sexual debut.

## Results

### Sample description

The sample of 1907 had an average age of 17 years (ranging from 14 to 24) and was 55% male (Table 1). Fewer than 4% were married or cohabitating, far lower than national averages for this age group [25]. Approximately one in five (410 adolescents) was sexually active, with about 50% of these reporting modern contraceptive use. Just under 3% reported being pregnant or making a girl pregnant.

**Table 1:** Facility and adolescent characteristics

	Mean/percentage (N)
***Facility characteristics (n = 69)***	
Type of healthcare facility	
Dispensary	88.4 (61)
Clinic or health care center	11.6 (8)
Medical personnel	
Total (mean number)	3.96
Any medical officers, assistant medical officers	8.7 (6)
Any pharmacists/pharm assistants	2.9 (2)
Any medical assistants/clinical officers/medical attendants	95.7 (66)
Any nurses/midwives	92.8 (64)
***Adolescent characteristics (n = 1907)***	
Age(mean years)	17.2 (1903)
Female	44.6 (849)
Education: some secondary	39.3 (747)
Marital status: single or never married	96.3 (1832)
Sexual debut	21.6 (410)
HIV testing, past year	36.6 (696)
HIV testing, lifetime	50.9 (971)
SRH visit past year	16.7 (318)
SRH visit lifetime	18.1 (345)
Health facility in village	66.7 (1270)

Over half the adolescents (971) reported a previous HIV test, with over a third (696) reporting that they had received a test in the past year (54% of adolescents who had sexually debuted and 32% of those who had never had sex; results not shown). Utilization of SRH services was much lower: only 18% (345) reported ever visiting a health facility for HIV/SRH services (76% of adolescents who had sexually debuted and 28% of those who had never had sex; results not shown).

Facility-level characteristics (*n* = 69) are given in Table 1. Most (88%) were classified as dispensaries, and would typically provide outpatient services. Only 12% were designated as clinics or health care centers, and thus may have additional in-patient services available [26]. On average, facilities employed four health care workers, most commonly nurses/midwives and medical attendants. No facilities reported having a doctor or social worker on staff.

### Availability of adolescent-friendly health services

We find that most adolescents (67%) resided in a village with at least one health facility (Table 1). On average, health facilities reported being open for adolescent HIV testing over 60 h per week (Table 2). Finally, over half the facilities considered adolescents’ ability to pay in determining fees.

**Table 2:** Availability of adolescent-friendly health services at the 69 health facilities surveyed

	Facilities % (N)
***Accessibility***	
Total hours open per week for adolescent HIV testing(mean)	61.2
Has flexible hours for adolescents	14.5 (10)
Staff participated in school meetings to inform parents/guardians about health services available to adolescents	33.3 (23)
Staff participated in community meetings to inform youth and other organizations about health services available for adolescents	49.3 (34)
Has communication support materials	17.4 (12)
Has a separate waiting room for adolescent patients	5.8 (4)
***Appropriate***	
Has a referral system	11.6 (8)
receives supervisory visits	18.8 (13)
***Equitable***	
Offers contraceptive counselling and services to all adolescents	91.3 (63)
Offers HIV services for all adolescents	100
***Effective***	
Has Policies/guidelines/procedures with regard to adolescents	37.7 (26)
Essential services available	
HIV testing/counseling	100
HIV treatment	94.2 (65)
Other STI testing/counseling	97.1 (67)
Gender-based violence services	43.5 (30)
Family planning/contraceptives	100

Staff promoted adolescent health services to adolescents, parents and teachers through community (49% of facilities) and school meetings (33%). A smaller proportion (17%) distributed support materials to parents and other community members on the value of providing health services to adolescents.

We find facilities were largely able to fulfill the SRH needs of adolescents. All facilities offered HIV testing and family planning services; the majority (97%) offered other STI testing, and only slightly fewer (94%) offered HIV treatment. Few facilities discriminated based on marital status: almost all (91%) offered contraceptive services regardless of marital status; and all facilities offered HIV testing regardless of marital status. However, less than half (44%) offered services for survivors of gender-based violence (GBV). Finally, only 12% of facilities had a system in place for referral and follow-up with adolescent clients.

Few facilities had structures in place to support the effective delivery of adolescent services. Only a third of health facilities reported that they had documents with policies, guidelines or procedures that were specific to adolescents. One in five received supportive supervisory visits related to adolescent friendly services from the Ministry of Health or other similar trainers.

Finally, adolescents who reported seeking SRH services were asked about their experiences to gauge acceptability (Table 3). Overall, adolescents found the staff friendly (98%), were comfortable asking questions (82%), and felt they received adequate answers to their questions about sexual and reproductive health (97%). The majority (91%) reported that the services were adequately confidential.

**Table 3:** Perceived quality of health services provided at last SRH visit (*N* = 318 adolescents)

***Acceptable***	Perceived quality
	% (N)
Adolescent felt comfortable asking SRH staff questions	81.8 (318)
Staff answered SRH questions adequately	97.3 (260)
Staff was friendly	97.5 (318)
SRH services were adequately confidential	91.2 (318)

### Determinants of HIV testing and SRH service uptake

Table 4 presents the risk ratios for lifetime HIV testing, past year HIV testing and SRH visits. Adolescents with a clinic located in their home village were more likely to report a past-year SRH visit (RR = 1.32, *p* < 0.05) and there was a trend towards greater lifetime HIV testing (RR = 1.13, *p* < 0.1). Adolescents who lived near more inclusive clinics, as measured by the provision of contraceptive regardless of marital status, were more likely to report lifetime HIV testing (RR = 1.22, *p* < 0.05) and a past-year SRH visit (RR = 1.50, *p* < 0.001). The latter association appears to be driven by adolescents who are sexually active and most in need of contraceptives (RR = 1.71, *p* < 0.1). Neither a convenient location nor an inclusive policy on contraception were predictive of past-year testing.

**Table 4:** Risk ratios predicting HIV testing and SRH visits by availability of AFHS characteristics

	HIV testing, lifetime	HIV testing, past year			SRH visit		
	Full sample	Full sample	Sexually debuted	Not sexually debuted	Full sample	Sexually debuted	Not sexually debuted
***Accessibility***							
Has health facility in the same village	1.13* [0.98;1.30]	0.99 [0.85;1.17]	1.00 [0.85;1.42]	1.00 [0.79;1.16]	1.32** [1.01;1.72]	1.32 [0.94;1.85]	1.36* [0.94;1.97]
Total hours open per week for adolescent HIV testing	1.00 [1.00;1.00]	1.00 [1.00;1.00]	1.00 [1.00;1.00]	1.00 [1.00;1.00]	1.00 [1.00;1.00]	1.00 [1.00;1.00]	1.00 [1.00;1.00]
Flexible hours for adolescents	1.10 [0.97;1.25]	1.17** [1.00;1.37]	1.28** [1.06;1.55]	1.11 [0.88;1.39]	1.20 [0.95;1.52]	1.32*** [1.02;1.72]	1.06 [0.69;1.61]
School meetings	1.08 [0.93;1.26]	1.02 [0.86;1.22]	1.16 [0.94;1.42]	0.99 [0.79;1.24]	1.03 [0.79;1.33]	1.06 [0.82;1.38]	1.07 [0.70;1.63]
Community meetings	1.13* [0.98;1.30]	1.15* [0.98;1.36]	1.19 [0.95;1.48]	1.13 [0.93;1.38]	1.11 [0.84;1.46]	1.27 [0.90;1.79]	0.97 [0.67;1.41]
Materials for community	1.13 [0.90;1.42]	1.19 [0.94;1.51]	0.87 [0.63;1.20]	1.30 [0.96;1.75]	1.16 [0.81;1.66]	0.95 [0.68;1.32]	1.34 [0.78;2.29]
Separate waiting room	1.01 [0.75;1.35]	1.07 [0.78;1.48]	0.91 [0.49;1.68]	1.12 [0.80;1.57]	0.99 [0.62;1.60]	1.17 [0.63;2.17]	0.89 [0.54;1.44]
***Appropriate***							
Referral system	1.16 [0.88;1.52]	1.28* [0.97;1.70]	1.22 [0.96;1.56]	1.26 [0.85;1.87]	1.44** [1.08;1.93]	1.56*** [1.21;2.01]	1.23 [0.64;2.37]
Supervisory visits	1.14 [0.95;1.37]	1.06 [0.85;1.32]	1.04 [0.73;1.48]	1.09 [0.87;1.38]	0.92 [0.64;1.33]	0.93 [0.62;1.37]	0.99 [0.61;1.62]
***Equitable***							
Contraceptives for all youth	1.22** [1.02;1.48]	1.10 [0.90;1.34]	1.18 [0.89;1.56]	1.05 [0.75;1.47]	1.50*** [1.17;1.93]	1.35 [0.86;2.12]	1.71* [0.99;2.96]
***Effective***							
Policies/guidelines/procedures	1.13* [0.99;1.29]	1.12 [0.95;1.31]	1.09 [0.86;1.38]	1.13 [0.93;1.37]	1.20 [0.93;1.54]	1.22 [0.90;1.65]	1.19 [0.81;1.74]
Essential services available							
HIV treatment	0.84 [0.67;1.05]	0.86 [0.68;1.08]	0.87 [0.72;1.04]	0.83 [0.61;1.30]	1.05 [0.69;1.59]	1.41 [0.65;3.05]	0.72 [0.41;1.30]
Other STI testing/counseling	0.84* [0.71;1.00]	0.70*** [0.61;0.80]	0.87 [0.61;1.25]	0.66*** [0.50;0.87]	0.78 [0.54;1.14]	3.19** [1.30;7.88]	0.34*** [0.27;0.43]
Gender-based violence services	1.03 [0.89;1.20]	1.05 [0.88;1.25]	1.20* [0.98;1.47]	1.01 [0.82;1.25]	1.06 [0.81;1.40]	1.41** [1.07;1.86]	0.87 [0.58;1.32]
N	1905	1901	409	1580	1905	410	1583

Each risk ratio represents a separate regression. All models adjust for age, education, gender, SES, marital status, sexual debut, type of facility, and total personnel

*p-value<0.1; ** *p* value<0.05; *** *p* value<0.01; Confidence intervals in brackets

Having a referral system in place was a strong determinant of SRH visits (RR = 1.44, *p* < 0.05), with some evidence of larger effect sizes among those who were sexually active, and demonstrated a trend with past-year HIV testing (RR = 1.28, p < 0.1). Among those adolescents who were sexually active only, the availability of GBV services was associated with an increased likelihood of both past-year HIV testing (RR = 1.20, *p* < 0.05) and SRH visits (RR = 1.41, *p* < 0.01).

We had hypothesized that the availability of other STI testing and counseling would be positively associated with both HIV testing and SRH visits. It did demonstrate a strong positive association with SRH visits (RR = 3.19, p < 0.05), but only for those adolescents who were sexually active. For those that had not yet sexually debuted, the association was negative (RR = 0.34, p < 0.01). It similarly demonstrated a negative relationship with both lifetime and past-year HIV testing. Additional models of past-year HIV testing that controlled for prior HIV testing yielded qualitatively similar results (not shown).

## Discussion

This study sought to describe the uptake of HIV testing and SRH services among adolescents in two regions of Tanzania, and relate such to the availability of AFHS. We found that about half the sample have ever received an HIV test, consistent with national reports on youth testing [14]. However, less than one in five reported seeking SRH services. Given the gap between those adolescents reporting HIV testing and those reporting SRH services, it would appear that adolescents are predominately being tested outside of public health facilities. This is consistent with data that shows adolescents want testing to be delivered outside health services (e.g., at community venues, their household, or through mobile testing) [8], and suggests that we may want to extend the AFHS framework to services outside of the healthcare realm. It may also reflect gaps in AFHS offered at local health facilities.

Critically, we find that AFHS have not been fully implemented. While Tanzania institutionalized AFHS standards over a decade ago, the guidelines are not being fully translated into practice at the local level. Less than 40% of facilities reported that they had guidelines for adolescent care, and fewer than one in five received supervision visits related to AFHS.

On a more positive note, adolescents who have sought SRH care in the past report staff were friendly, respected privacy, and could answer their questions. Moreover, health facilities appear to be geographically and financially accessible to adolescents. Most essential SRH services, including HIV testing and treatment, STI testing, and contraceptives, are almost universally available. However, we find that GBV services are uncommon: they are available at only two out of five health facilities. This is a notable gap, given that almost a third of girls will experience sexual abuse by the time they reach age 18 [29] and the vast majority will experience GBV as adults [30]. Importantly, the availability of GBV services was associated with both elevated HIV testing and SRH visits. Scaling up this essential, basic service could further promote HIV testing, in addition to providing much needed support for survivors of GBV.

To optimize resources, we need to know what other components of AFHS best facilitate HIV testing and SRH service utilization and should be prioritized. A cross-sectional study in Burundi, for example, found that a youth-designated check-in, community outreach, and flexible hours were associated with adolescents’ use of SRH services [31]. Many characteristics related to staffing, such as training, were not predictive. In our study, adolescents were more likely to seek HIV testing and SRH services when health facilities were in close proximity, had a referral system, offered GBV services, and were more inclusive, as measured by the provision of contraceptive regardless of marital status. Unlike the Burundi study, we did not find that opening hours were important, though this may reflect that facilities already offered flexible hours. In our study, facilities were open an average of 60 h per week for HIV testing.

An important dimension of AFHS is equity. We expected that facilities with policies that were more inclusive would be associated with greater service use. One potential barrier is marital status. In Tanzania and elsewhere, a substantial proportion of providers say they would not provide unmarried adolescents with SRH services [32–34]. In our sample, over 90% of facilities stated that they offered contraceptive counselling and services to all adolescents, irrespective of marital status. While high, this means that almost 10% continue to discriminate by marital status. Moreover, our findings suggest that this policy depresses both HIV testing and SRH service utilization. We note that we do not measure provider bias or attitudes directly, which may be present even in clinics with favorable policies. Thus, ensuring policies are in line with AFHS standards is a critical first step; it may be that additional training is also warranted [33].

A final and critical aspect of equity is whether adolescents of all ages can access services. Evidence shows that lowering the age of consent can encourage testing, and current WHO guidelines recommend that countries revise their policies to reduce age-related barriers to HIV testing and care [12]. The present study was done at a time when the provision of HIV testing was still restricted by age of majority. At the end of 2019, Tanzania took steps to reduce this legal barrier, dropping the age of consent to 15. As younger adolescents gain access to essential HIV testing, it will be important to re-examine whether the AFHS identified in this study retain the same relationship with service outcomes.

Past research has shown that community support for adolescent SRH services is a key facilitator of uptake, and may be more important than facility characteristics [35]. Community and parental opposition can be a barrier to obtaining adolescent services, even more so when access depends on parental consent. Fostering community support is thus a cornerstone of AFHS. We expected greater uptake of services among adolescents whose local facility promoted the benefits of adolescent testing. However, we did not observe any impact from organized school or community meetings, or from providing materials to communicate with the value of adolescent health services.

The lack of significant impact of community support may mean that many AFHS elements are not essential to ensure access to HIV testing and SRH services. However, it may also mean that these elements, such as the community outreach referenced above, are being poorly implemented [24]. A major limitation of this study is that we do not measure quality or dose. Other studies have shown that focusing on quality improvements (through training, supervision, and support) increases testing knowledge and may encourage HIV testing (e.g., [36]). In our study, however, supervisory visits from the Ministry of Health or similar organizations was not associated with HIV testing outcomes.

### Strengths and limitations

The major strength of this study is its ability to identify which elements of AFHS are most critical to HIV testing and SRH uptake in government run facilities by linking facility-level data from government run facilities with individual reports from adolescents. The AFHS policy is intended to have national coverage, but is not fully implemented. Our design takes advantage of the natural variation between health facilities. The AFHS characteristics identified as beneficial –offering GBV services, offering referral services, and having policies that promote inclusivity –are by definition already being implemented by public facilities. They are not yet universal, and could feasibly be adopted by others.

A major limitation of this study is that we cannot link adolescents to specific facilities where they receive care. We instead match them to the facility in closest proximity to their home. However, qualitative work done in conjunction with the broader evaluation from which this study is drawn has shown that youth may prefer health facilities that are further away in order to avoid stigma [37]. Other studies have shown that they prefer private clinics [38]. Moreover, HIV testing could have occurred in non-traditional settings, such as schools or home. This could introduce measurement error, and reduce the strength of observed associations. Moreover, presence of friendly, confidential staff has emerged as one of the strongest determinants of adolescent engagement in HIV testing and SRH [39–41]. However, the lack of linkage between adolescent reports and facility data means that we are unable to examine whether positive perceptions of staff were associated with greater uptake of subsequent services. Finally, we rely on behavioral self-report for HIV testing and SRH utilization. Self-report may be artificially low [16] due to recall bias; or it may be inflated due to social desirability bias. The high levels of perceived confidentiality and communication are consistent with other studies on AFHS, however we caution that the lack of variation across studies may suggest adolescents are reluctant to criticize providers [42].

## Conclusion

In this study we link novel data from government-run health facilities and adolescent surveys to examine the association between uptake of HIV and SRH services and AFHS in two regions in Tanzania. We found that the following characteristics of services were associated with increased uptake among adolescents: proximity, had a referral system, offered GBV services, and inclusivity. As existing evidence has highlighted, implementation is key, and our study highlighted gaps in full implementation of AFHS standards. Continued efforts are needed to strengthen services to align with guidelines. This may lead to stronger observed associations between quality of AFHS and uptake of services among adolescents, and subsequently, better HIV testing and treatment, fewer unplanned pregnancies, and healthier birth intervals.

## Data Availability

Data analysed for this study are not publicly available but may become available, subject to government approval, after completion of the impact evaluation.

## Appendix

### Study Design and Sampling Information

The impact evaluation (from which wave 2 survey data were used for the current study) uses a cluster randomized control trial (cRCT) design to examine impacts of an adolescent focused ‘Cash Plus’ intervention layered on top of Tanzania’s flagship social protection program, the Productive Social Safety Net (PSSN), implemented by the Tanzania Social Action Fund (TASAF). Ujana Salama complements the PSSN with a package of adolescent-focused interventions to strengthen productive, human and health capital to promote sustainable and healthy livelihoods that increase resilience, well-being and empowerment.

In this cRCT design, 130 clusters (communities) from two pilot administration areas of TASAF (in the Iringa and Mbeya regions of Tanzania) were randomized into two control and treatment arms, and randomization was stratified by district and village size (large v. small villages). During a public randomization event with district-level government officials, village names in each district were written on a piece of paper and placed into one of two hats (large villages v. small villages). District leaders then chose names from the hat and read them aloud, while the study coordinator wrote the village names down in order until all villages had been withdrawn. The top (first) half of the list was assigned heads and the second half tails. District officials completed a coin toss for each list (small and large villages) to assign villages to treatment.

All adolescents living in TASAF households in communities sampled within the eligibility age range were targeted for interviews, and sample size was determined based on power calculations of key outcomes from the study’s theory of change. Randomization took place in July 2017, after implementation of the baseline surveys (April – June 2017). The on-going evaluation is a multi-year, longitudinal, mixed-method study comprised of baseline (2017), wave 2 (2018), wave 3 (2019), and wave 4 (2021) surveys.

## Abbreviations

AFHS: Adolescent friendly health services
cRCT: Clustered randomized controlled trial
GBV: Gendered based violence
LMIC: Low- and middle-income countries
MOHSW: Tanzania’s Ministry of Health and Social Welfare
PSSN: Productive Safety Net Programme
SRH: Sexual and reproductive health
STI: Sexually transmitted infections
TASAF: Tanzania Social Action Fund
WHO: World Health Organization
